# The Antioxidant and Antiaging Properties of Maillard Reaction Products Derived From Dipeptide Lys–Leu in *Caenorhabditis elegans*

**DOI:** 10.1002/fsn3.71427

**Published:** 2026-01-14

**Authors:** Yaqi Jia, Issei Yokoyama, Yusuke Komiya, Jun Nagasao, Keizo Arihara

**Affiliations:** ^1^ Department of Animal Science, School of Veterinary Medicine Kitasato University Aomori Japan; ^2^ Department of Animal and Marine Bioresource Sciences, Graduate School of Agriculture Kyushu University Fukuoka Japan

**Keywords:** antiaging, antioxidant activity, bioactive peptides, *C. elegans*, Maillard reaction products, *sod‐3*

## Abstract

The Maillard reaction, a nonenzymatic browning reaction that occurs between amines and carbonyl groups during food processing and cooking, generates products with antioxidant activity. Dipeptides composed of leucine and lysine (Leu–Lys and Lys–Leu) are frequent sequences in a variety of food proteins. Previously, we demonstrated that these dipeptides exhibit antioxidant activity and extend the lifespan of the nematode *Caenorhabditis elegans* ( *C. elegans* ). Although the Maillard reaction can improve peptide bioactivity, its effects on Leu–Lys and Lys–Leu remain unclear. Therefore, we investigated the antioxidant activity of Maillard reaction products (MRPs) derived from these dipeptides in vitro and their effects on the aging process in *C. elegans* . The antioxidant activity of Leu–Lys was unaffected by the Maillard reaction. By contrast, MRPs generated from Lys–Leu exhibited the highest antioxidant activity after 2 h of heat treatment with glucose. In wild‐type *C. elegans* , the administration of Lys–Leu MRPs extended lifespan under both normal and oxidative stress conditions and improved motility with aging. In addition, Lys–Leu MRPs reduced the accumulation of reactive oxygen species and increased the mRNA expression of an antioxidant‐related gene (*sod‐3*). However, lifespan extension by Lys–Leu MRPs was not observed in *sod‐3* and *daf‐16* mutants. These findings suggest that Lys–Leu MRPs extend the lifespan of *C. elegans* via the insulin/insulin‐like growth factor signaling pathway.

## Graphical abstract

Lys–Leu MRPs heat‐treated for 2 h exhibited high antioxidant activity in vitro. A treatment with Lys–Leu MRPs extends the lifespan and health span of *C. elegans* by regulating ROS levels through their inherent antioxidant activity and the IIS pathway.

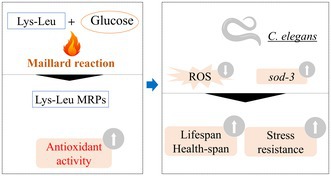

## 1 Introduction

Aging is an inevitable process associated with improved living standards and extended lifespans. It is characterized by a gradual decline in physiological functions and an increased susceptibility to diseases. A considerable number of theories have been advanced to explain the underlying mechanisms of aging, with oxidative stress emerging as a central factor. Oxidative stress arises when there is an imbalance between the production of reactive oxygen species (ROS) and the body's antioxidant defense capacity (Sies and Cadenas 1985; Ziada et al. 2020). ROS are natural byproducts of cellular metabolism. Moderate levels of ROS act as signaling molecules and play a crucial role in various cellular functions. However, excessive ROS have been demonstrated to cause oxidative damage to cellular components, including DNA, proteins, and lipids (Ortuño‐Sahagún et al. 2014; Liguori et al. 2018). The resulting cellular dysfunction contributes to the decline in organ function and overall health. Moreover, as organisms age, the efficiency of their antioxidant defense systems declines, resulting in increased oxidative stress. This increased oxidative stress, in turn, has been demonstrated to accelerate the aging process and to significantly contribute to the development of various age‐related diseases, including cancer, diabetes, and cardiovascular disorders (Harman 1956; Benz and Yau 2008; Chen et al. 2012).

Antioxidants play a crucial role in mitigating aging. They effectively reduce oxidative damage and preserve normal cellular function and structure (Kozlov et al. 2024; Liu et al. 2018). Numerous studies have shown that antioxidants have the capacity to diminish oxidative stress and decelerate cellular aging (Ghzaiel et al. 2021; Rusu et al. 2019; Sánchez‐Rodríguez et al. 2016). Moreover, antioxidants can enhance cellular antioxidant capacity through the activation of intracellular defense mechanisms, such as the insulin/insulin‐like growth factor (IIS) signaling pathway (Tabibzadeh 2021). Antioxidants are widely found in a variety of foods, including fruits, vegetables, and processed foods. Therefore, strategies to mitigate oxidative stress, such as maintaining a diverse diet and taking supplements, are crucial for extending the lifespan of older adults and improving their quality of life.

The Maillard reaction is a significant chemical reaction involved in food processing and cooking. This reaction occurs when amino acids and peptides react with carbonyl compounds, such as reducing sugars, during heating. The Maillard reaction is a complex chemical reaction that is divided into three stages: initial, intermediate, and final stages, each of which produces different products. The Maillard reaction products (MRPs) include various chemicals such as odorants and brown pigments, which significantly affect the color and flavor of processed foods (Ohata et al. 2018; Yokoyama 2025). Furthermore, MRPs offer health‐promoting benefits because of their potent antioxidant activities (Kitts 2021; Yokoyama et al. 2021; Miyaki et al. 2025). Thus, the application of the Maillard reaction in food processing and development positively influences both sensory and functional properties.

In this study, we utilized the nematode *Caenorhabditis elegans* ( *C. elegans* ) as a model organism to evaluate the antioxidant and antiaging effects of MRPs. *C. elegans* is a well‐established model in aging research because of its short lifespan, ease of handling, and transparency, which facilitates microscopic observation (Tissenbaum 2015; Sulston et al. 1983; Zhang et al. 2020). It also possesses essential organs similar to those of humans, including the epidermis, muscles, nervous system, digestive tract, and reproductive organs (Zhang et al. 2020). *C. elegans* can be cultured on agar plates with *Escherichia coli* ( *E. coli* ) at 20°C, with a generation time of 3–4 days and an average lifespan of approximately 3 weeks. Importantly, approximately 60%–80% of *C. elegans* genes share homology with humans, and they possess conserved signaling pathways, such as the IIS pathway, making them ideal models for aging studies (Shaye and Greenwald 2011). Previous reports have indicated that administering food‐derived antioxidants can inhibit the production of ROS associated with aging, thereby prolonging the lifespan of *C. elegans* (Yazaki et al. 2011; Sadowska‐Bartosz and Bartosz 2014).

Previously, we showed that dipeptides composed of leucine and lysine (Lys–Leu and Leu–Lys, respectively) exhibited in vitro antioxidant activity, prolonged lifespan, and reduced intracellular ROS levels in *C. elegans* (Yokoyama et al. 2023). Since these sequences are commonly found in food proteins, Lys–Leu and Leu–Lys are generated in fermented foods, such as cured meats (Zhu et al. 2017) and fermented soybeans (Sato et al. 2018). Because the Maillard reaction can enhance the antioxidant activity of peptides (Arihara et al. 2021), we hypothesized that this reaction contributes to the improved biological properties of Leu–Lys and Lys–Leu. Therefore, the objective of this study was to compare the in vitro antioxidant activities of MRPs derived from Leu–Lys and Lys–Leu. Additionally, the present study investigated the effects of MRPs on the lifespan of *C. elegans* .

## 2 Materials and Methods

### 2.1 Materials

The following reagents were obtained from FUJIFILM Wako Pure Chemical Co.: 2′‐deoxy‐5‐fluorouracil (FUdR), 4% paraformaldehyde (PFA) solution, and hypoxanthine. D‐glucose, KH_2_PO_4_, and NaCl were procured from Kanto Chemical Co. 2‐Methyl‐6‐p‐methoxyphenylethynyl‐imidazopyrazinone (MPEC) was purchased from ATTO Co. (Osaka, Japan). The dipeptides Lys–Leu and Leu–Lys, which were used for the antioxidant activity assays, *C. elegans* N2 Bristol lifespan assays, intracellular ROS measurements, and oxidative stress resistance assays, were synthesized by SCRUM Inc. (Tokyo, Japan) and GenScript (Tokyo, Japan).

### 2.2 Generation of Maillard Reaction Products

The dipeptides Lys–Leu and Leu–Lys (final concentration: 10 mg/mL) and D‐glucose (0.1 M) were mixed in 0.5% (w/v) sodium carbonate buffer. The mixtures were then heated at 90°C for 2, 4, and 6 h, respectively, using a dry thermos unit (DTU‐2CN, TAITEC Co., Japan). The reaction mixtures were immediately cooled on ice and referred to as the MRPs (Leu–Lys/Lys–Leu MRPs). In addition, a solution consisting of the dipeptides and glucose was prepared without heating (designated as Leu–Lys/Lys–Leu, unheated). All solutions were stored at −30°C until needed.

### 2.3 Antioxidant Activity Assay

The assessment of antioxidant activity was conducted through the implementation of a chemiluminescence assay, which served to quantitate the capacity of the sample to scavenge superoxide radicals (Gulcin 2020). The hypoxanthine‐xanthine oxidase system gives rise to superoxide radicals which then interact with MPEC to generate chemiluminescence. The presence of antioxidant substances in the sample leads to the elimination of superoxide radicals, consequently resulting in reduced chemiluminescence values (Sassetti et al. 2021). Subsequently, following our previous method (Zain et al. 2024), the measurement of chemiluminescence was conducted employing a luminescence‐PSN (AB‐2200, ATTO Co.). For the assay, 10 μL of the sample was mixed with 180 μL of KH_2_PO_4_ buffer (0.1 M) and MPEC (final concentration 300 μM) mixture solution, 60 μL of xanthine oxidase solution (0.005 U/mL), and 50 μL of 0.72 mM hypoxanthine solution (0.54 g of KH_2_PO_4_, 0.8 g of EDTA‐2Na, 0.08 g of NaOH, and 0.002 g of hypoxanthine). The measurement of chemiluminescence was carried out for 20 s. Distilled water was utilized as the negative control. The luminescence inhibition rate, which is indicative of antioxidant activity, was calculated via the following formula:

$$\text{Antioxidant activity}\;\left(\%\right)=\left[\left(A–B\right)/A\right]\times 100$$

*A*: luminous amount of negative control, *B*: luminous amount of tested sample.

Subsequently, the relative values were calculated using the unheated sample (0 h) as the basis.

### 2.4 Culture and Age Synchronization of *C. elegans*

The wild‐type strain of *C. elegans* N2 Bristol was provided by the *Caenorhabditis* Genetics Center (University of Minnesota, Minneapolis, MN, USA). The mutant strains *skn‐1* (*tm4241*), *daf‐16* (*mu86*), and *sod‐3* (*tm670*) were obtained from the National Bio Resource Project (NBRP) *C. elegans* . *C. elegans* was cultivated on nematode growth medium (NGM) plates that had been seeded with *E. coli* OP50 at 20°C for 4 days (Brenner 1974). To obtain age‐synchronized nematodes, gravid adults were subjected to bleaching (Porta‐de‐la‐Riva et al. 2012). Specifically, gravid adults on the NGM plate were collected in a 15 mL test tube and washed using 0.16 M NaCl (S‐buffer). Then, age‐synchronized larvae were obtained as previously described (Yokoyama et al. 2021). Subsequent to the one‐night hatch, the larvae were collected and utilized for all subsequent experiments.

### 2.5 Lifespan Assay

In order to investigate the impact of the MRPs on lifespan, an assay was conducted based on our previous report using the *C. elegans* system (Yokoyama et al. 2023). Age‐synchronized L1 larvae were moved to 24‐well plates, which contained heat‐killed *E. coli* OP50 and liquid medium. The cultures were incubated at 20°C with continuous shaking at 130 rpm. Once the nematodes reached adulthood, FUdR (final concentration 0.5 mg/mL) was added to each well to stop the production of offspring. Each test solution was added to the wells (final concentration 10 mg/mL). Day 0 was marked as the day when the test solution was added, and nematodes were continuously cultured at 20°C with 130 rpm shaking. The survival count was measured under a microscope every 3 days until all *C. elegans* had perished. The experiment was performed at least three times.

### 2.6 Movement Assay

As previously described (Yokoyama et al. 2021), a movement assay was carried out for the assessment of the health span of *C. elegans* . Age‐synchronized L1 larvae were cultured in a liquid medium, which had heat‐killed *E. coli* OP50 at 20°C with shaking at 100 rpm for 3–4 days. The test solutions with a final concentration of 10 mg/mL and FUdR (final concentration 0.5 mg/mL) were then added to the cultures, which were incubated for an additional 3, 7, or 11 days. On the day of the experiment, the nematodes were moved to NGM plates without *E. coli* OP50. Using an inverted microscope (SZX7; OLYMPUS, Tokyo, Japan), the number of body bends was recorded within 30 s. Each treatment group had at least 30 nematodes that were examined.

### 2.7 Measurement of Intracellular ROS

The nematodes' ROS levels were measured with 2′,7′‐dichlorofluorescein diacetate (H_2_DCF‐DA; Invitrogen, Carlsbad, CA, USA). As mentioned above, the age‐synchronized adult nematodes were incubated for an additional 4 days with the test solution (final concentration 10 mg/mL) and FUdR (final concentration 0.5 mg/mL). Subsequently, the nematodes were incubated with H₂DCF‐DA solution (final concentration 50 μM) for a duration of 60 min in a dark environment. Following the incubation, the nematodes underwent another washing with S‐buffer and were then fixed with 4% PFA solution for 10 min. After being processed, the nematodes were placed on 2% agarose pads for clear images to be obtained. A BZ‐X800 fluorescence microscope (Keyence) was utilized for fluorescence microscopy. The quantification of fluorescence intensity through the Green Fluorescent Protein filter (excitation: 450–490 nm; emission: 500–550 nm) was carried out by ImageJ software. For each group, fluorescence intensity was measured in at least 20 nematodes.

DHE reagents (FUJIFILM Wako Pure Chemical Co., Osaka, Japan) were used to determine intracellular superoxide radical levels in the nematodes. The adult nematodes were incubated with DHE solution (final concentration 5 μM) at 20°C for 30 min in a dark environment. After that, the nematodes were fixed with 4% PFA solution and placed on agarose pads. Using ImageJ software, fluorescence intensity through the Texas Red filter (excitation: 540–580 nm; emission: 595–670 nm) was quantified in no less than 20 nematodes per group.

### 2.8 Evaluation of Oxidative Stress Resistance

The impact of MRPs on lifespan in the presence of oxidative stress was evaluated as previously stated (Yokoyama et al. 2023). Adult nematodes were cultured for 4 days after adding test solution (final concentration 10 mg/mL) and FUdR (final concentration 0.5 mg/mL). Subsequently, 270 μL of paraquat solution (final concentration 5 mM) and 30 μL of heat‐killed *E. coli* OP50 were added to each well. The number of nematodes was enumerated and marked as Day 0. Nematodes were observed every 2 days until all nematodes had perished.

### 2.9 Quantitative Real‐Time PCR

For gene expression analysis, changes in mRNA expression after Lys–Leu MRPs treatment were measured as previously stated (Yokoyama et al. 2021). Approximately 5000 L1 larvae that were age‐synchronized were cultured in liquid medium, which had heat‐killed *E. coli* OP50 in 250 mL culture flasks at 20°C until they reached adulthood. The nematodes were cultured for 4 days after the addition of the test solution (final concentration 0.5 mg/mL) and FUdR (final concentration 0.5 mg/mL), with continuous shaking at 130 rpm. TRIzol reagent (Invitrogen) was utilized for the extraction of total RNA. SuperScript III reverse transcriptase (Invitrogen, final concentration 8 U/μL) and oligo (dT) 12–18 primers (Invitrogen, final concentration 2.8 μg/μL) were used for cDNA synthesis, in accordance with the manufacturer's guidelines. The Applied Biosystems StepOne Plus system (Applied Biosystems, Waltham, MA, USA) was utilized for performing quantitative PCR with PowerUp SYBR Green Master Mix (Thermo Fisher Scientific, Waltham, MA, USA). Table 1 contains the primers utilized in this study. The *cdc‐42* was employed as the housekeeping gene for *C. elegans* .

**TABLE 1:** Sequences of primer sets used for RT‐qPCR in *C. elegans* .

Gene	Forward primer sequence (5′‐3′)	Reverse primer sequence (5′‐3′)	Amplicon size (bp)
*cdc‐42*	CTGCTGGACAGGAAGATTACG	CTCGGACATTCTCGAATGAAG	111
*daf‐16*	CCAGACGGAAGGCTTAAACT	ATTCGCATGAAACGAGAATG	149
*sod‐3*	ATCTACTGCTCGCACTGCTT	TTTCATGGCTGATTACAGGTT	128
*ctl‐1*	CGGATACCGTACTCGTGATGAT	CCAAACAGCCACCCAAATCA	185
*skn‐1*	GGTCTCCGTTGGCGTGATGATC	CTGGTGGATGCTCGGTGAGTATTG	103
*gst‐4*	GATGCTCGTGCTCTTGCTG	CCGAATTGTTCTCCATCGAC	158
*gcs‐1*	TGTTGATGTGGATACTCGGTG	TGTATGCAGGATGAGATTGTACG	125

### 2.10 Statistical Analysis

Each assay was carried out independently three times. Each group of nematodes must contain no fewer than 30 individuals. Data are presented as the mean ± standard error of the mean (SEM). The log‐rank test was employed to analyze statistical significance in lifespan assays, with *p* values indicating significance (*p* < 0.05). Concurrently, the hazard ratio (HR) was calculated using the Cox proportional hazards model. For other experiments, the Tukey–Kramer comparison was conducted with *p* values reported to assess significance (*p* < 0.05). Excel‐Toukei ver. 7.0 (Social Survey Research Information Co. Ltd., Tokyo, Japan) was utilized for all statistical analyses.

## 3 Results

### 3.1 Antioxidant Activity of the MRPs Generated From Dipeptides

**FIGURE 1:**
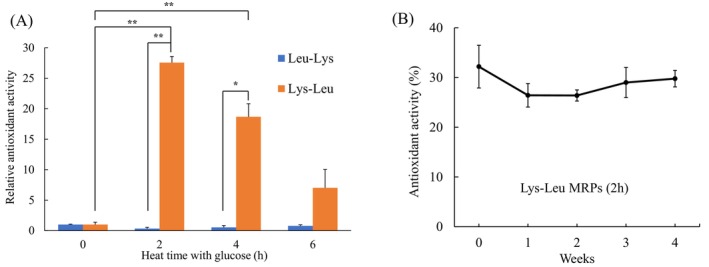
Antioxidant activities of the Maillard reaction products from dipeptides (Lys–Leu and Leu–Lys) against superoxide radicals. (A) Relative antioxidant activities with heating time. (B) Changes in the activity of Lys–Leu MRPs with storage time. Data are presented as mean ± SE (*n* = 3). Significant differences were determined by two‐way ANOVA followed by the Tukey–Kramer multiple comparison test (*p < 0.05; **p < 0.01).

The antioxidant activities of the dipeptide–glucose mixtures against superoxide radicals were measured after heating for 0, 2, 4, and 6 h (Figure 1A). The antioxidant activity of the Leu–Lys mixture was found to be unaffected by heating. By contrast, after heating with glucose for 2 and 4 h, the antioxidant activity of the Lys–Leu mixture, i.e., Lys–Leu MRP, was found to increase significantly in comparison with the Lys–Leu unheated (0 h) mixture (*p* < 0.01). The Lys–Leu MRPs exhibited the highest antioxidant activity after 2 h of heat treatment with glucose. To assess their stability, the antioxidant activity of Lys–Leu MRPs was examined after storage. The activity was retained for at least 4 weeks at 20°C (Figure 1B).

### 3.2 Effect of Lys–Leu MRPs on the Lifespan and Health Span of Wild‐Type *C. elegans*

**FIGURE 2:**
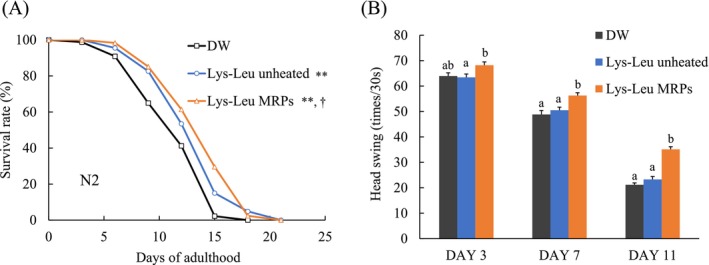
Effect of the Maillard reaction products on the lifespan and motility of *C. elegans* . (A) Changes in survival rate, expressed as the mean of three independent experiments. Statistical analysis was performed using the log‐rank test (*p < 0.05 vs. DW; †*p* < 0.05 vs. unheated Lys–Leu). (B) Changes in motility of wild‐type *C. elegans* . Data are presented as mean ± SE (*n* = 135 ± 20). Statistical analysis was conducted using the Tukey–Kramer multiple comparison test. Different letters indicate significant differences at each day (a‐b, *p* < 0.05). DW, distilled water; Lys–Leu, unheated Lys–Leu mixed with glucose; Lys–Leu MRPs, Lys–Leu Maillard reaction products heated for 2 h.

According to the results of the antioxidant activity assay, the lifespan of *C. elegans* N2 (wild‐type) was measured in response to treatment with unheated Lys–Leu and Lys–Leu MRPs (heated for 2 h). In comparison with the control group, both unheated Lys–Leu and Lys–Leu MRPs demonstrated a significant extension in the lifespan of the nematodes, with Lys–Leu MRPs being more effective (Figure 2A). The figure also demonstrates that Lys–Leu MRP effectively extended median lifespan. The average and maximum lifespans, and HR of all groups are summarized in Table 2. Specifically, the average lifespan increased by approximately 13% in the Lys–Leu group and by approximately 20% in the Lys–Leu MRPs group compared with that in the control. For maximum lifespan, both unheated and heated samples exhibited an extension of approximately 3 days. All HR values were less than 1, and Lys–Leu and Lys–Leu MRPs reduced the hazard of death. Lys–Leu MRPs demonstrated a significant reduction.

**TABLE 2:** Effect of the Maillard reaction products on *C. elegans* lifespan.

Groups	Average lifespan (days)	Effect (%)	Median lifespan (day)	Maximum lifespan (day)	Hazard ratios
DW	11.94 ± 1.01^a^	—	12	18	—
Lys–Leu unheated	13.54 ± 0.80^b^	+13.38	15	21	0.81
Lys–Leu MRPs	14.31 ± 0.86^c^	+19.78	15	21	0.60

*Note:* Data are presented as mean ± SE. The assay was independently repeated three times. (DW, *n* = 119; Lys–Leu unheated, *n* = 153; Lys–Leu MRPs, *n* = 140). Significant differences were analyzed using the Tukey–Kramer test (a‐c, *p* < 0.05). Hazard ratio was calculated using the Cox proportional hazards model.

Abbreviation: DW, distilled water.

As nematodes reach the young adult stage, they exhibit intense and rapid body movement. However, with aging, their movement speed gradually decreases. Movement indicators, such as head swings amplitude and frequency, have also been demonstrated to reflect health status, with a decrease in these indicators usually indicating a shortened health span (Hsu et al. 2009; Pincus and Slack 2010). In this experiment, head swings frequency was used as an indicator of health span. The results are shown in Figure 2B. On day 3, a significant difference was observed in the number of head swings between the Lys–Leu MRP group and the Lys–Leu unheated group (*p* < 0.05). However, such significance was not observed compared to the control group. On days 7 and 11, both the Lys–Leu unheated and control groups showed a marked decline in motility, whereas the Lys–Leu MRP group also exhibited a decrease; its head swing frequency remained significantly higher than that of both the control and unheated Lys–Leu groups. This finding suggested that Lys–Leu MRPs attenuated the age‐related decline in physical activity.

### 3.3 Effect of Lys–Leu MRPs on ROS Levels in *C. elegans*

**FIGURE 3:**
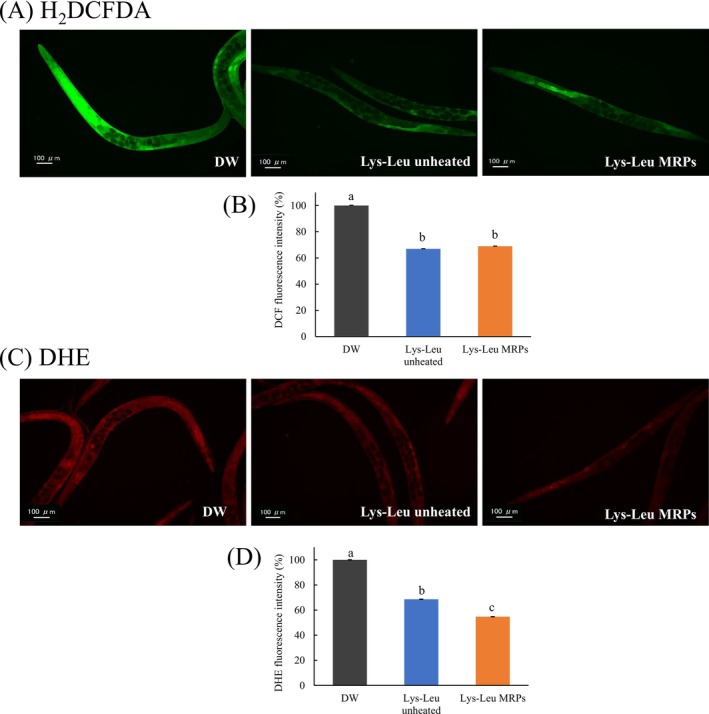
Quantitative analysis of intracellular reactive oxygen species (ROS) and superoxide radical levels. (A) Representative images of H_2_DCF‐DA‐stained *C. elegans* ; (B) Fluorescence quantification of intracellular ROS; (C) Representative images of DHE‐stained *C. elegans* ; (D) Fluorescence quantification of superoxide radicals. Statistical analysis was conducted using the Tukey–Kramer multiple comparison test. Different letters indicate significant differences (a‐c, *p* < 0.05). DW, distilled water; Lys–Leu, unheated Lys–Leu mixed with glucose; Lys–Leu MRPs, Lys–Leu Maillard reaction products heated for 2 h.

To evaluate the effects of unheated Lys–Leu and Lys–Leu MRPs on oxidative stress, we stained intracellular ROS in *C. elegans* using H_2_DCF‐DA and DHE. H_2_DCF‐DA produces green fluorescence upon oxidation by various ROS, allowing quantitative assessment of intracellular ROS levels through fluorescence intensity measurements. Figure 3A shows representative images of nematodes stained with H_2_DCF‐DA. Both the Lys–Leu unheated and Lys–Leu MRPs groups exhibited a significant reduction in fluorescence intensity (Figure 3B, *p* < 0.05). However, no significant difference was observed between the Lys–Leu unheated and Lys–Leu MRPs groups.

DHE, a fluorescent probe used to detect superoxide radicals, produces red fluorescence in the presence of intracellular superoxide. Figure 3C shows representative images of DHE‐stained nematodes. Compared with the control group, the fluorescence intensity was significantly reduced by 32% and 46% in the unheated Lys–Leu and Lys–Leu MRPs treatments, respectively (Figure 3D, *p* < 0.05). Moreover, a significant difference was observed between the Lys–Leu unheated and Lys–Leu MRPs groups (*p* < 0.05).

### 3.4 Effect of Lys–Leu MRPs on the Lifespan of *C. elegans* Under Oxidative Stress

**FIGURE 4:**
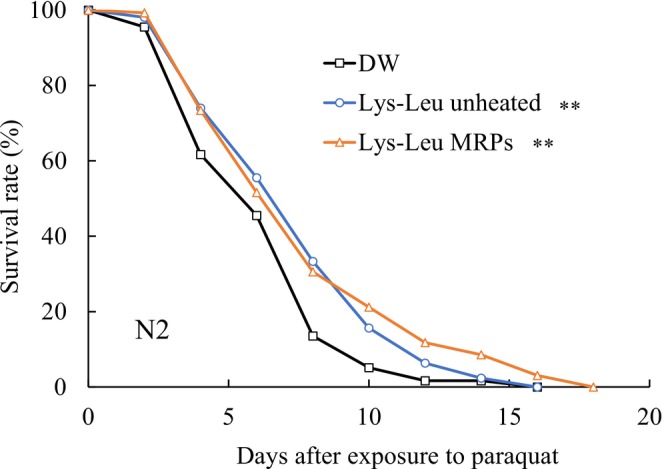
Effect of the Maillard reaction products on the lifespan of *C. elegans* exposed to 5 mM paraquat. Changes in survival rate are expressed as the mean of three independent experiments. Statistical analysis was performed using the log‐rank test (**p < 0.05). DW, distilled water; Lys–Leu, unheated Lys–Leu mixed with glucose; Lys–Leu MRPs, Lys–Leu Maillard reaction products heated for 2 h.

Nematodes were subjected to paraquat‐induced oxidative stress, and their lifespans were measured. The lifespan of the Lys–Leu unheated group was found to be significantly extended relative to the control group. A similar trend was observed in the Lys–Leu MRP group (Figure 4). As shown in Table 3, the average lifespan of the Lys–Leu unheated group increased by approximately 18%, while that of the Lys–Leu MRPs group increased by 23% compared with observations in the control. The maximum lifespan of the Lys–Leu MRPs group was prolonged by 2 days. Additionally, Lys–Leu MRPs mitigate the hazard of death under oxidative stress.

**TABLE 3:** Effect of the Maillard reaction products on oxidative stress in *C. elegans* .

Groups	Average lifespan (days)	Effect (%)	Median lifespan (day)	Maximum lifespan (day)	Hazard ratios (vs. control)
DW	6.48 ± 0.23^a^	—	4	16	—
Lys–Leu unheated	7.65 ± 0.23^b^	+18.08	4	16	1.04
Lys–Leu MRPs	7.99 ± 0.29^b^	+23.23	4	18	0.84

*Note:* Data are presented as mean ± SE. The assay was independently repeated three times. (DW, *n* = 227; Lys–Leu unheated, *n* = 188; Lys–Leu MRPs, *n* = 152). Significant differences were analyzed using the Tukey–Kramer test (a‐b, *p* < 0.05). Hazard ratio was calculated using the Cox proportional hazards model.

Abbreviation: DW, distilled water.

### 3.5 Expression of Antioxidant‐Related Genes

**FIGURE 5:**
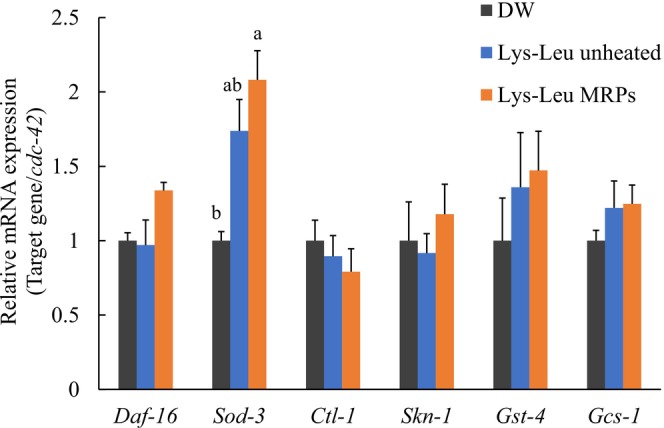
mRNA expression of antioxidant‐related genes in *C. elegans* . Data are presented as mean ± SE from three independent experiments. Statistical analysis was conducted using the Tukey–Kramer multiple comparison test. Different letters indicate significant differences (a‐b, *p* < 0.05). DW, distilled water; Lys–Leu, unheated Lys–Leu mixed with glucose; Lys–Leu MRPs, Lys–Leu Maillard reaction products heated for 2 h.

The mRNA expression of antioxidant‐related genes in *C. elegans* was examined via RT‐qPCR, and the results are shown in Figure 5. Treatment with Lys–Leu MRPs resulted in a significant upregulation in the expression of the *sod‐3* gene (*p* < 0.05). Although there was no statistically significant difference compared with the control group, unheated Lys–Leu also increased *sod‐3* expression (*p* = 0.050). By contrast, the mRNA expression levels of *ctl‐1, skn‐1, gst‐4*, and *gcs‐1* remained unchanged.

### 3.6 Effect of Lys–Leu MRPs on the Lifespan of *C. elegans* Mutants

**FIGURE 6:**
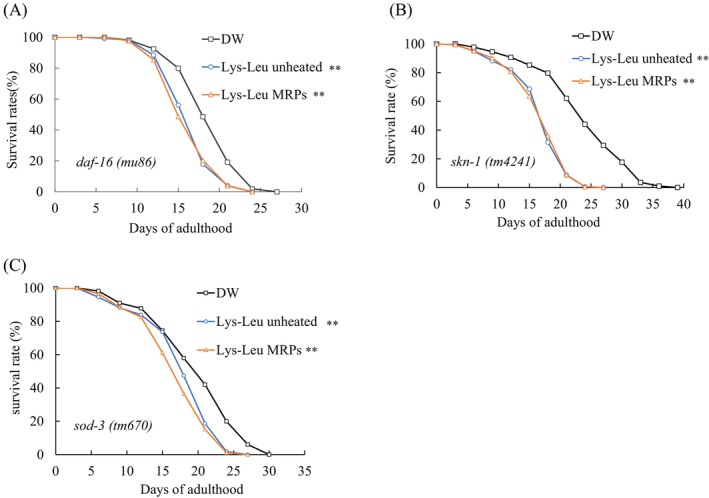
Effect of the Maillard reaction products on the lifespan of *C. elegans* mutants. (A) Lifespan of *daf‐16* mutants treated with Lys–Leu and Lys–Leu MRPs; (B) Lifespan of *skn‐1* mutants; (C) Lifespan of *sod‐3* mutants. Changes in survival rate are expressed as the mean of three independent experiments. Statistical analysis was performed using the log‐rank test (**p < 0.01). DW, distilled water; Lys–Leu, unheated Lys–Leu mixed with glucose; Lys–Leu MRPs, Lys–Leu Maillard reaction products heated for 2 h.

To investigate the molecular mechanisms underlying the effects of Lys–Leu MRPs on longevity and health in *C. elegans* , we measured the lifespans of three mutant strains: *skn‐1, daf‐16*, and *sod‐3*. These three mutant strains exhibit deletions in their respective genes. If the mutants' lifespan shows no significant change, this suggests that the treatment may extend lifespan through this gene. If the lifespan of the mutants exhibits no significant change, it implies that the treatment may extend lifespan via this gene. Our results indicated that both unheated Lys–Leu and Lys–Leu MRPs failed to extend the lifespan of these mutants compared with the control (Figure 6A–C). The average and maximum lifespans of the mutant strains are presented in Table 4. These results suggest that the lifespan extension effects of both unheated Lys–Leu and Lys–Leu MRPs require functional *daf‐16*, *skn‐1*, and *sod‐3*.

**TABLE 4:** Effect of Lys–Leu MRPs on the lifespan of the *C. elegans* mutant.

Groups	Average lifespan (days)	Effect (%)	Median lifespan (day)	Maximum lifespan (day)	Hazard ratios (vs. control)
*skn‐1 (tm4241)*					
DW	24.18 ± 0.56^a^	—	21	39	—
Lys–Leu unheated	17.23 ± 0.60^b^	−28.74	18	27	1.82
Lys–Leu MRPs	17.25 ± 0.28^b^	−28.63	18	27	1.78
*daf‐16 (mu86)*					
DW	19.21 ± 0.12^a^	—	18	27	—
Lys–Leu unheated	16.81 ± 0.26^b^	−12.47	18	24	1.85
Lys–Leu MRPs	16.68 ± 0.42^b^	−13.19	15	24	1.75
*sod‐3 (tm670)*					
DW	20.13 ± 0.33^a^	—	21	30	—
Lys–Leu unheated	18.24 ± 0.68^b^	−9.37	18	27	1.05
Lys–Leu MRPs	17.45 ± 0.16^b^	−13.31	18	27	1.22

*Note:* Data are presented as mean ± SE. The assay was independently repeated three times. (*skn‐1*, DW, *n* = 180; Lys–Leu unheated, *n* = 176; Lys–Leu‐ MRPs, *n* = 173. *daf‐16*, DW, *n* = 166; Lys–Leu unheated, *n* = 167; Lys–Leu‐ MRPs, *n* = 167. *sod‐3*, DW, *n* = 164; Lys–Leu unheated, *n* = 163; Lys–Leu MRPs, *n* = 172). Significant differences were analyzed using the Tukey–Kramer test (a‐b, *p* < 0.05). Hazard ratio was calculated using the Cox proportional hazards model.

Abbreviation: DW, distilled water.

## 4 Discussion

MRPs exhibit significant antioxidant activity, scavenge free radicals, and hold potential for the development of functional foods with health benefits. Their antioxidant activity is influenced by thermal conditions, substrate type, and other variables (Chen et al. 2019; Cao et al. 2022). Different combinations of amino compounds and reducing sugars affect the antioxidant properties of MRPs (Kitts 2021). Before heat treatment, the antioxidant activity of Leu–Lys was higher than that of Lys–Leu, consistent with the results of our previous study (Yokoyama et al. 2023). However, after heat treatment with glucose, Lys–Leu exhibited higher antioxidant activity than that of Leu–Lys. Lysine contains both α‐ and ε‐amino groups, which contribute to its high reactivity in the Maillard reaction (Golon et al. 2014). In peptides, the hydrophobic side chain (isobutyl) of leucine significantly influences the reactivity of lysine (Mennella et al. 2006). Lys–Leu, with leucine at the N‐terminus, exhibits high reactivity in the Maillard reaction. Therefore, antioxidant MRPs may be more readily generated through heat treatment. Substances responsible for antioxidant activity in MRPs primarily include melanoidins, heterocyclic intermediates, and phenolic compounds (Cao et al. 2022; Shakoor et al. 2022; Bolchini et al. 2025). Melanoidins are mixtures composed of various macromolecular compounds with extremely complex structures. The complete structures of these compounds have not yet been fully elucidated due to the difficulty inherent in identifying them (Wang et al. 2011). The antioxidant activity of MRPs generally increases with extended heating times (Chen et al. 2019). However, in the present study, Lys–Leu MRPs exhibited reduced antioxidant activity after 4 h of heat treatment. According to a previous report, the antioxidant activity of MRPs generated from histidine and glucose decreased after heating at 120°C for over 30 min (Yilmaz and Toledo 2005). Therefore, it is hypothesized that antioxidative MRPs generated from Lys–Leu, such as melanoidins, may undergo degradation or structural rearrangement during prolonged heating. As Lys–Leu MRPs heated for 2 h exhibited the highest antioxidant activity among all samples, they were selected for subsequent *C. elegans* experiments.

Antioxidants can extend the lifespan of *C. elegans* (Lin et al. 2019; Wang et al. 2018). An increase in ROS levels contributes to oxidative stress, which accelerates aging and reduces motility (Yang et al. 2024). Our findings demonstrated that Lys–Leu MRPs significantly reduced ROS levels in *C. elegans* , thereby attenuating the age‐related decline in motility. Motility serves as a key indicator for assessing health span and aging in *C. elegans* . The attenuation of motility decline indicates that Lys–Leu MRPs have the potential to improve the health span of *C. elegans* . Additionally, Lys–Leu MRPs significantly reduced the accumulation of intracellular ROS, particularly superoxide radicals, compared with observations in the Lys–Leu unheated group. This difference may be attributed to the enhanced in vitro superoxide radical scavenging capacity resulting from heat treatment. Paraquat, an herbicide, is commonly used to induce oxidative stress in *C. elegans* (Bora et al. 2021). Although paraquat reduced the average lifespan of treated nematodes, Lys–Leu MRPs significantly prolonged lifespan compared with observations in the control. A plausible explanation is that Lys–Leu MRPs activate the endogenous antioxidant defense system, thereby eliminating excess ROS generated by paraquat.

The IIS pathway plays a central role in regulating the lifespan of *C. elegans* . It modulates metabolism, stress responses, and longevity through multiple downstream effectors (Murphy and Hu 2013). The *daf‐16* gene is a key transcription factor in the IIS pathway and promotes longevity by enhancing stress resistance. The *sod‐3* and *ctl‐1* are downstream target genes of *daf‐16*. The *sod‐3* encodes a mitochondrial manganese superoxide dismutase (Mn‐SOD), an enzyme that catalyzes the dismutation of superoxide radicals into oxygen and hydrogen peroxide, thereby mitigating cellular damage induced by ROS (Hunter et al. 1997). Decreased IIS pathway activity activates *daf‐16*, which enters the cell nucleus and initiates transcription of the *sod‐3* and *ctl‐1* genes, upregulating their expressions. Studies indicate that increased mRNA levels of the *sod‐3* gene extend the lifespan of *C. elegans* (Honda and Honda 1999; Zečić and Braeckman 2020). The *ctl‐1* gene encodes a catalase that protects cells from oxidative stress by catalyzing the decomposition of hydrogen peroxide. CTL‐1 functions in concert with genes including *daf‐16* and *sod‐3* to coordinately regulate oxidative stress responses and lifespan in *C. elegans* (Zhou et al. 2021). MRPs derived from lysine and glucose upregulate *daf‐16*, *sod‐3*, and *ctl‐1 expression* (Yokoyama et al. 2021). Sugar‐derived advanced glycation end products (AGEs) have been shown to extend lifespan through activation of DAF‐16 (Papaevgeniou et al. 2019). We demonstrated that Lys–Leu MRPs have the capacity to scavenge superoxide radicals. Consequently, *daf‐16* and its downstream genes, *sod‐3* and *ctl‐1*, were selected for gene expression analysis. In the present study, Lys–Leu MRPs significantly increased *sod‐3* mRNA expression, suggesting that Lys–Leu MRPs enhance antioxidant capacity, alleviate oxidative stress, and extend lifespan in *C. elegans* via upregulation of *sod‐3*. We additionally evaluated the impact of Lys–Leu and Lys–Leu MRPs on the lifespan of *daf‐16* and *sod‐3* mutants. Neither unheated Lys–Leu nor Lys–Leu MRPs extended the lifespan of *daf‐16* (*mu86*) or *sod‐3* (*tm670*) mutants. These findings indicate that the lifespan‐extending effects of both forms are mediated through the IIS pathway.

Furthermore, *skn‐1*, a key Nrf‐family transcription factor in *C. elegans* , regulates the expression of cytoplasmic antioxidant genes (Blackwell et al. 2015). In some contexts, *skn‐1* and *daf‐16* synergistically regulate antioxidant gene expression, enhancing antioxidant defenses and lifespan. For example, under hypoxia–reoxygenation conditions, the activation of both *skn‐1* and *daf‐16* promotes the expression of their target genes, mediating resistance to anoxic starvation and extending lifespan (Siswanto et al. 2024). *Gst‐4* and *gcs‐1* are downstream target genes of *skn‐1*. Increased expression of both genes helps to maintain the intracellular redox balance and thereby extends the lifespan of nematodes. Therefore, *skn‐1* and its downstream target genes *gst‐4* and *gcs‐1* were selected. The results indicated that the expression levels of the *skn‐1*, *gst‐4*, and *gcs‐1* genes remained unchanged. To further investigate whether the lifespan‐extending effects of Lys–Leu and Lys–Leu MRPs are related to *skn‐1*, the lifespan of *skn‐1* mutant (*tm4241*) was measured. The lifespan of the mutants was not extended by treatment with unheated Lys–Leu or Lys–Leu MRPs. The *skn‐1* (*tm4241*) mutant exhibits a slightly shortened lifespan under normal conditions compared with the wild‐type (Tullet et al. 2017). Lys–Leu MRPs reduced the lifespan of the *skn‐1* (*tm4241*) mutant but did not upregulate *skn‐1* gene expression in wild‐type nematodes.

Finally, high glucose concentrations (100 mM) significantly shorten the lifespan of *C. elegans* (Alcántar‐Fernández et al. 2018). Considering the final concentration of 100 mM glucose used in the present study, it is plausible that the glucose in the unheated Lys–Leu contributed to the lifespan reduction observed in all mutants. Furthermore, the presence of residual unreacted glucose in Lys–Leu MRPs is postulated as a potential factor contributing to the observed reduction in lifespan of mutants. In our previous study, Lys–Leu exhibited low antioxidant properties but significantly extended the lifespan of wild‐type *C. elegans* (Yokoyama et al. 2023). These findings suggest that Lys–Leu in unheated solution may directly induce lifespan extension mediated by *daf‐16* and *skn‐1*, which outweighs the negative effects of glucose.

There are, however, two major limitations of the present study that should be addressed in future research. First, we did not investigate the antiaging effects of Lys–Leu or its MRPs in the absence of glucose. Although antioxidative MRPs are widely present in the reaction mixture, the antiaging effects of the major antioxidative MRPs derived specifically from Lys–Leu and glucose should be further examined. Second, although Lys–Leu MRPs failed to extend the lifespan of *skn‐1* mutants, they also did not affect the expression of antioxidant genes, such as *gst‐4* and *gcs‐1*, which are involved in the SKN‐1/Nrf2 pathway. Further investigation of upstream genes in the *skn‐1* signaling cascade (e.g., *nsy‐1*, *sek‐1*, and *pmk‐1*) and more comprehensive gene analysis are necessary to elucidate the mechanism by which Lys–Leu MRPs may influence longevity via several signaling pathways.

## 5 Conclusion

**FIGURE 7:**
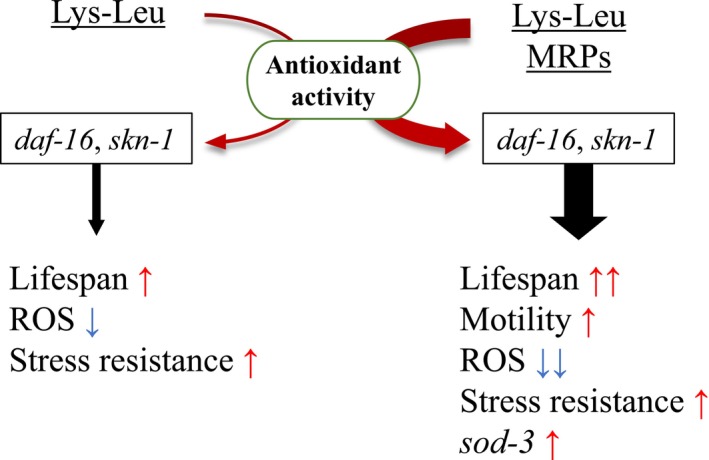
Proposed mechanism by which Lys–Leu MRPs affect lifespan and signaling pathways in *C. elegans* . *daf‐16*, forkhead box protein O; Lys–Leu MRPs, Lys–Leu Maillard reaction products; ROS, reactive oxygen species; *skn‐1*, transcription factor skinhead‐1; *sod‐3*, superoxide dismutase 3.

In this study, we demonstrated that MRPs generated from Lys–Leu and glucose exhibited higher antioxidant activity than that of Leu–Lys MRPs. Lys–Leu MRPs significantly extended the lifespan of wild‐type *C. elegans* and improved health span. Additionally, Lys–Leu MRPs effectively reduced intracellular ROS levels and enhanced tolerance to oxidative stress. These effects were further supported by the upregulation of the antioxidant gene *sod‐3*. Treatment with Lys–Leu MRPs reduced the lifespan of *sod‐3* and *daf‐16* mutants, suggesting that MRPs modulate lifespan through the IIS signaling pathway (Figure 7). Our findings indicate that Lys–Leu may serve as a functional food ingredient with antioxidant and antiaging properties when subjected to the Maillard reaction.

## Author Contributions

**Yaqi Jia:** investigation, visualization, and writing – original draft. **Issei Yokoyama:** conceptualization, methodology, validation, supervision, project administration, funding acquisition, writing – review and editing. **Yusuke Komiya, Jun Nagasao:** methodology and reviewing draft. **Keizo Arihara:** conceptualization, supervision, funding acquisition, writing – review and editing, validation, project administration.

## Funding

This work was supported by the Japan Society for the Promotion of Science (JP21H02348 and JP22KJ2827).

## Ethics Statement

The authors have nothing to report.

## Conflicts of Interest

The authors declare no conflicts of interest.

## Data Availability

The data that support the findings of this study are available from the corresponding author upon reasonable request.
